# Effects of Extrusion Technology on Physicochemical Properties and Microstructure of Rice Starch Added with Soy Protein Isolate and Whey Protein Isolate

**DOI:** 10.3390/foods13050764

**Published:** 2024-03-01

**Authors:** Xiaofei Liu, Xiangxiang Zhao, Chunmin Ma, Ming Wu, Qiqi Fan, Yu Fu, Guang Zhang, Xin Bian, Na Zhang

**Affiliations:** 1 College of Food Engineering, Harbin University of Commerce, Harbin 150028, China; liuxiaofei72@163.com (X.L.); 18845649291@163.com (X.Z.); chunmin_ma@163.com (C.M.); mingle1219@outlook.com (M.W.); fanqiqi990@163.com (Q.F.); rczkzg@163.com (G.Z.); bianbian1225@163.com (X.B.); 2 College of Food Science, Southwest University, Chongqing 400715, China; fuy987@swu.edu.cn

**Keywords:** soy protein isolate, whey protein isolate, extrusion technology, microstructure, physicochemical properties

## Abstract

In order to improve the retrogradation of rice starch (RS) and the quality of rice products, soy protein isolate (SPI), whey protein isolate (WPI), and rice flour were mixed and further extruded into mixed flour. The physicochemical properties and morphology of starch of extruded rice flour (ERS) and starch of extruded mixtures of SPI, WPI, and rice flour (SPI-WPI-ERS) were analyzed. The distribution of amylopectin chain length, molecular weight, microstructure, crystallinity, short-range ordered structure, pasting properties, and thermodynamic properties of RS, ERS, and SPI-WPI-ERS were measured. The results showed that, compared with rice starch, the proportion of long-chain starch, total starch content, and molecular weight were decreased in ERS and SPI-WPI-ERS, but the proportion of short-chain and amylose content was increased. The short-range order structure was destroyed. The water absorption of ERS and SPI-WPI-ERS was much higher than rice starch at 55 °C, 65 °C, and 75 °C, but lower than that of rice starch at 95 °C. Therefore, the retrogradation characteristics of SPI-WPI-ERS were improved. The setback of rice starch products was reduced and the setback of SPI-WPI-ERS was lower than that of ERS. Overall, the retrogradation of rice starch was delayed by adding exogenous protein and extrusion technology, and the application range of rice flour in staple food products was broadened.

## 1. Introduction

Rice is grown in more than 100 countries and regions. It is the third-largest cereal crop in the world after wheat and maize [1], which is the main source of energy for more than half of the population [2]. Rice contains nutrients such as starch and protein, and is the most suitable raw material for making gluten-free food due to its unique flavor, low allergy, and good processing characteristics [3,4]. The main source of carbohydrates in rice is starch, which accounts for 90% of the dry weight of rice [5]. Rice starch, characterized by easy digestion, light taste, small particles, and easy acceptance by consumers [6], is widely used in the field of food and medicine [7].

However, natural rice starch has some limitations, such as a low water absorption and water solubility, and serious retrogradation during storage [8]. Ai et al. [9] showed that, with the extension of storage time, rice cakes became harder due to retrogradation of the rice starch, thereby significantly shortening the shelf life. Therefore, how to control and delay the retrogradation of rice starch, effectively improve the storage quality, and prolong the shelf life of rice products have become the key issues to be solved [10,11]. Currently, there are several methods for starch modification, such as spray drying, drum drying, high hydrostatic pressure, thermomechanical processing, and extrusion cooking followed by drying [12]. Among these techniques, the extrusion method avoids the traditional chemical modification of starch, which is more acceptable to consumers [13]. Wet swellable starches and protein materials are physically modified by a unique combination of high temperature, high pressure, and shear forces after extrusion treatment. The gelatinization of starch was changed, and then the setback of natural rice starch decreased [14].

During the process of extrusion, protein is unfolded, rearranged, hydrolyzed, and cross-linked with other components (e.g., starch), which further delays the retrogradation of rice starch [15]. Moreover, many starch-based extrudates have low nutritional value, while exogenous protein can improve the nutritional density of extrudates [16]. Soybean protein isolate (SPI) has a high amino acid content and contains many nutritional factors, and has good gelation and a low sensitivity [17]. Whey protein isolate (WPI) is a by-product of the dairy industry, which can be widely used in the food field to improve the nutritional value of food and can also form a gel paste or rigid matrix through interaction with a starch system [18]. A mixture of texturized whey protein isolate, corn starch, and pea starch could improve the viscosity, breakdown, setback, and hardness of starch [19]. In recent years, a number of research works have focused on the combined effects of adding a type of exogenous protein and extrusion on starch properties [20,21]. However, the effect of SPI, WPI, and rice flour blends on the properties of rice starch is still unclear, and the underlying mechanism among SPI, WPI, and rice starch is not clear enough.

Therefore, this study investigated the influence of the extrusion process on the retrogradation characteristics of starch in rice noodles with SPI and WPI. The microstructure of rice starch and percentage of retrogradation were investigated using scanning electron microscopy (SEM), a polarizing microscope (PLM), rapid viscosity analysis (RVA), and thermal properties. In addition, the crystallinity and short-range order structure of retrograded starch were investigated using X-ray diffraction (XRD) and Fourier transform infrared (FTIR) spectroscopy. These studies contributed to understanding the change in rice starch and SPI-WPI-rice starch during extrusion and providing a theoretical basis for the quality adjustment of rice products and the development of modified rice starch products.

## 2. Materials and Methods

### 2.1. Materials

The rice flour used in this study was provided by Jiaxiang Yongsheng Food Factory. The SPI and WPI were purchased from Guzhiwei Biotechnology Co., Ltd. (Shenzhen, China). The rice flour, WPI, and SPI (WPI: SPI = 1:3) were mixed evenly according to the protein addition of 9.52%. The mixed rice flour was crushed in a high-speed multi-function crusher for 3 min (800 C, Yongkang Hongtaiyang Electromechanical Co., Ltd., Yongkang, China), followed by passing through a 120-mesh sieve. Then, the raw materials were obtained.

### 2.2. Preparation of Extruded Samples

The content of water in the raw materials (120-mesh) was adjusted to 28% (dry basis), then the raw materials were extruded three times using a twin-screw extruder (DSE-25, German Brabender Co., Ltd., Duisburg, Germany) after mixing [22]. The parameters during extrusion were set. The feed rate was 35 r/min, the screw speed was 122 r/min, and the temperatures of zone Ⅰ, zone Ⅱ, zone Ⅲ, zone Ⅳ, and zone Ⅴ were 50, 60, 70, 80, and 127 °C, respectively. The extruded rice flour and extruded mixture of SPI, WPI, and rice flour were obtained. The extruded samples were dried in a hot air oven (DHG-9145A, Shanghai Yiheng Scientific Instrument Co., Ltd., Shanghai, China) at 40 °C and the moisture content was kept at 7%, followed by passing through 120-mesh sieves after crushing using a high-speed multi-function crusher (800 C, Yongkang Hongtaiyang Electromechanical Co., Ltd., Yongkang, China) for subsequent experiments’ samples [23].

### 2.3. Preparation of RS, ERS, and SPI-WPI-ERS

The RS, ERS, and SPI-WPI-ERS were soaked in a 0.4% NaOH solution at a mass ratio of 1:10 for 24 h in a beaker, centrifuged at 4000 r/min for 20 min using a high-speed centrifuge (NP-H2-16K, Ningbo Beijiao instrument technology group Co., Ltd., Ningbo, China), and the supernatant and yellow precipitate were discarded. The rest of the precipitate was washed with distilled water and centrifuged again until yellow precipitate was completely removed to obtain wet starch. The obtained wet starch was dried in a hot air oven (DHG-9145A, Shanghai Yiheng Scientific Instrument Co., Ltd., Shanghai, China) at 40 °C for 24 h and passed through a 120-mesh sieve for detection.

### 2.4. Molecular Structure of RS, ERS, and SPI-WPI-ERS

#### 2.4.1. Amylopectin Chain Length Distribution of RS, ERS, and SPI-WPI-ERS

RS, ERS, and SPI-WPI-ERS (10 mg) were dissolved in 5 mL of distilled water in a boiling water bath (HH-6, Shanghai Lichen Bangxi Instrument Technology Co., Ltd., Shanghai, China) for 60 min to prepare gelatinized samples. After cooling to room temperature, NaN_3_ (10 μL 2% *w*/*v*), acetate buffer (50 μL, 0.6 mol/L, pH 4.4), 0.5% (*w*/*v*) sodium borohydride solution, and isoamylase (10 μL, 1400 U) were added to 2.5 mL gelatinized samples, and placed at 37°C for 24 h. The mixture (600 μL) was put into a centrifuge tube and dried under nitrogen at room temperature. Then, it was dissolved in NaOH (30 μL, 1 mol/L) for 60 min, and the solution was diluted with 570 μL of distilled water, followed by centrifugation at 12,000 r/min for 5 min. Eventually, the supernatant was collected. The chain length distributions of RS, ERS, and SPI-WPI-ERS were determined by a Thermo ICS5000 ion chromatography system (ICS5000+, Thermo Fisher Scientific, Waltham, MA, USA).

#### 2.4.2. Total and Amylose Content of RS, ERS, and SPI-WPI-ERS

The total and amylose contents of RS, ERS, and SPI-WPI-ERS were determined according to the American Association of Cereal Chemists (AACC) Approved Method 61-03.01 [24].

#### 2.4.3. Molecular Weight of RS, ERS, and SPI-WPI-ERS

RS, ERS, and SPI-WPI-ERS (5 mg) were thoroughly mixed with 5 mL of DMSO solution containing LiBr (0.5% *w*/*w*) (DMSO/LiBr) and heated at 80 °C for 3 h. The average molecular weight (Mw), the number average molecular weight (Mn), and polydispersity index (Mw/Mn) of RS, ERS, and SPI-WPI-ERS were measured by a gel permeation chromatography-differential multi-angle laser light scattering system, the liquid phase system was U3000 (Thermo, Waltham, MA, USA), and the differential detector was OptilabT-rex (Wyatt technology, Goleta, CA, USA). The laser light scattering detector was DAWNHELEOS II (Wyatt technology, Goleta, CA, USA), λ = 663.7 nm.

#### 2.4.4. XRD Measurements of RS, ERS, and SPI-WPI-ERS

The crystallinity of RS, ERS, and SPI-WPI-ERS was determined by an X-ray diffractometer (D8 Advance, German Brook Co., Ltd., Shanghai, China). RS, ERS, and SPI-WPI-ERS were scanned over an angle range of 5–50° (2θ range) at room temperature. The target voltage was 40 kV, the current was 30 mA, the scanning speed was 5°/min, and the step was 0.02°. The crystallinity of RS, ERS, and SPI-WPI-ERS was analyzed using MDI Jade 6 software.

#### 2.4.5. FTIR Spectroscopy of RS, ERS, and SPI-WPI-ERS

The lyophilized RS, ERS, and SPI-WPI-ERS were mixed with KBr and scanned using an infrared spectrometer (PerkinElmer UATR Two, Perkin Elmer Enterprise Management Co., Ltd., Shanghai, China) with a wavelength from 4000 cm^−1^ to 400 cm^−1^. The spectra were analyzed by monitoring the changes in various chemical bonds in RS, ERS, and SPI-WPI-ERS.

#### 2.4.6. Micromorphology of RS, ERS, and SPI-WPI-ERS

The micromorphology of RS, ERS, and SPI-WPI-ERS was observed by SEM (S-3 400N, Hitachi, Tokyo, Japan) at ×1500 and ×200, respectively. After starch fixation and dehydration, the starch was replaced once by 100% ethanol and tert-butanol according to the volume ratio of 1:1, then replaced twice by pure tert-butanol. RS, ERS, and SPI-WPI-ERS were freeze-dried for 4 h. After gold plating, the microstructure of starch was observed by SEM at an operating voltage of 2 kV.

RS, ERS, and SPI-WPI-ERS (100 mg) were separately weighed, and the birefringence pattern of the starch granules was observed using a biological photo microscope (BX43, Olympus, Tokyo, Japan) assisted by polarized light.

### 2.5. Physical Properties of RS, ERS, and SPI-WPI-ERS

#### 2.5.1. Water Solubility Index (WSI) and Water Absorption Index (WAI) of RS, ERS, and SPI-WPI-ERS

The WSI and WAI of RS, ERS, and SPI-WPI-ERS were determined according to the AACC Approved Method 56-10.02 and the method of Paul et al. [25].

#### 2.5.2. RVA of RS, ERS, and SPI-WPI-ERS

The pasting properties of RS, ERS, and SPI-WPI-ERS were detected by RVA (2194617-TMB, Perten Instruments Co., Ltd., Stockholm, Sweden), 3.0 g of sample was transferred into a sample tube, and 25.0 mL of distilled water was added into the tube. RS, ERS, and SPI-WPI-ERS were heated from 50 °C to 95 °C, held at 95 °C for 5 min, then cooled to 50 °C and held at 50 °C for 5 min. The peak viscosity (PV), trough viscosity (TV), break down (BD) viscosity, final viscosity (FV), and setback (SB) viscosity of RS, ERS, and SPI-WPI-ERS were recorded during this process [26].

#### 2.5.3. Rheological Measurement of RS, ERS, and SPI-WPI-ERS

RS, ERS, and SPI-WPI-ERS were prepared by RVA and placed on a rheometer platform (MCR302e, Anton Paar Trading Co., Ltd., Shanghai, China). The test probe diameter of the rheometer was set to 25 mm, the distance between the two plates was 1 mm, and silicone oil was applied to the edge of the fixture to prevent water evaporation. After pressing the probe, RS, ERS, and SPI-WPI-ERS were equilibrated at 25 °C for 3 min to eliminate the deformation, which was caused by the extrusion of the probe, and were scanned between 0.1 and 20 Hz at a 1% strain amplitude. The variations in storage modulus (G′), loss modulus (G″), and phase angle (δ) were obtained from the tests, conducted in triplicates.

#### 2.5.4. Thermal Properties of RS, ERS, and SPI-WPI-ERS

RS, ERS, SPI-WPI-ERS (4 mg), and distilled water (8 µL) were sealed in stainless steel pans (Perkin Elmer Instruments LLC, Shelton, CT, USA) and equilibrated for 24 h at 4 °C in a refrigerator. The measuring conditions of the differential scanning calorimeter (PerkinElmer DSC4 000, Platinum Elmer Instruments Inc., Hopkinton, MA, USA) were as follows. The scanning temperature was 20–200 °C, then cooled from 200 °C to 20 °C, the scanning rate was 10 °C/min, the flow rate was 20 mL/min, and the protective gas was nitrogen. The onset (T_0_), peak (T_P_), and conclusion (Tc) gelatinization temperatures and gelatinization enthalpy (∆H) of starch were determined from the gelatinization curve.

### 2.6. Statistical Analysis

All analyses were performed in triplicates, and all results are expressed as mean ± SD. All data were analyzed for variance using SPSS 26.0 to assess differences between means. The Fisher least significant differences test was used to calculate the means with their 95% confidence intervals. All figures were plotted using Origin 9.0.

## 3. Results and Discussion

### 3.1. The Amylopectin Chain Length Distribution of RS, ERS, and SPI-WPI-ERS

**Figure 1:**
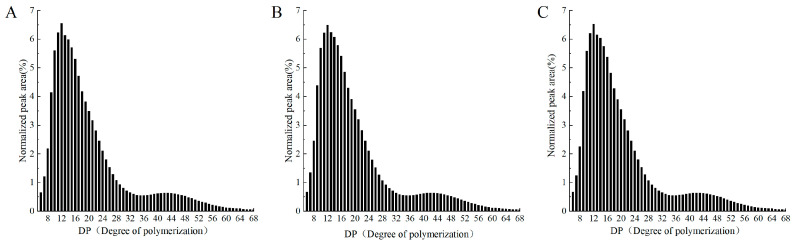
Normalized HPAEC-PAD chromatograms in amylopectin branch chain length distribution. (**A**) RS, (**B**) ERS, and (**C**) SPI-WPI-ERS.

A standardized HPAEC-PAD chromatogram of the amylopectin chain length distribution is shown in Figure 1. It can be seen from the figure that the highest peak value was DP11-13. The distributions of the amylopectin chain lengths of RS, ERS, and SPI-WPI-ERS exhibited the same trend. With an increase in the polymerization degree, the relative percentage of the normalized peak area firstly increased and then decreased. The results were similar to the conclusion of Jeong et al. [27] that the highest peak of rice starch with different contents of amylose was located in DP11-13.

**Figure 2:**
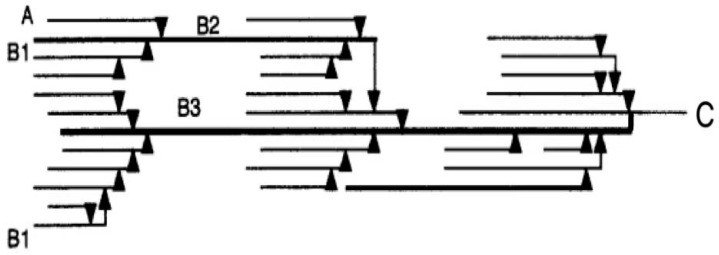
The models for the structure of amylopectin. (**A**) Short chain; (**B_1_**) medium-long chain; (**B_2_**) longer chain; (**B_3_**) long chain; (**C**) main-chain.

The models for the structure of amylopectin are shown in Figure 2. The length of the starch chain branch was divided into four parts. The A chain (6 ≤ DP ≤ 12) was the shortest, and it was connected to the B chain or the C chain through an ɑ-1,6 bond at the reducing terminal, which was called an outer chain. According to the length and number of clusters, the B chains were divided into B_1_ chains (13 ≤ DP ≤ 24, medium–long chain), B_2_ chains (25 ≤ DP ≤ 36, longer chain), and B_3_ chains (DP ≥ 37, long chain) [28].

The information on the chain length distributions of amylopectin in RS, ERS, and SPI-WPI-ERS is listed in Table 1. The proportions of amylopectin A, B_1_, B_2_, and B_3_ in rice starch were 26.63%, 49.97%, 11.18%, and 11.54% respectively. Compared with rice starch, the proportions of B_2_ chains and B_3_ chains in the amorphous region of ERS decreased (*p* < 0.05), and the proportions of short-chain A and B_1_ increased (*p* < 0.05). Liu et al. [12] also found that the maximum degradation occurred on the amylopectin molecule and the glycosidic bonds near the branching point of α-(1 → 6) or α-(1 → 4) amylopectin were prone to shear degradation during extrusion treatment. The change in the amylopectin length distribution of SPI-WPI-ERS was not more significant than that of ERS, which may have been due to the addition of SPI and WPI to reduce the degradation of rice starch after extrusion. The long chain of amylopectin promoted the formation of hydrogen bonds in the starch retrogradation process, and the short chain of amylopectin destroyed the stability of the crystalline lamellar structure [29]. The above results show that the degradation of starch increased after extrusion, which led to the degradation of amylopectin long chains into amylopectin short chains and amylose, and amylopectin short chains (6 **≤** DP **≤** 12) showed a negative relationship with retrogradation behavior, thus reducing the retrogradation performance of rice starch [30].

### 3.2. The Starch Content and Molecular Weight Distribution of RS, ERS, and SPI-WPI-ERS

The results of the total starch content, amylose content, and molecular weight analysis of RS, ERS, and SPI-WPI-ERS are summarized in Table 2. Starch was a kind of polymeric carbohydrate, polysaccharide composed of a single type of sugar units, and its molecular weight distribution was also an important parameter to characterize the molecular chain length of polymers [31].

The total starch content, Mn, and Mw of ERS and SPI-WPI-ERS were slightly lower than those of rice starch, but the content of amylose was higher than that of rice starch. The average molecular weight of the starch was reduced by extrusion, which further confirmed that extrusion leads to the degradation of starch molecules and refines the molecular size. The results indicated that the high temperature, high pressure, and high shear destroyed the original molecular structure of starch, and part of amylopectin was cut into amylose, which inhibited the rearrangement of starch molecules. Hence, the total starch content and starch molecular weight were reduced. The present results are consistent with the data of amylopectin length distribution mentioned above. The amylose content of SPI-WPI-ERS was less than that of ERS, and the total starch content was higher than that of ERS. The addition of WPI and SPI reduced the decomposition of starch by extrusion (*p* < 0.05). With the increased protein concentration, the starch granules of SPI-WPI-ERS were surrounded by protein, so this structure inhibited the starch swelling and release of amylose [32]. Mw/Mn was an important parameter to describe the molecular weight of starch [33]. The ratio of Mw/Mn increased, which resulted in a wider molecular weight distribution, the composition of the polymer became more complex, and the retrogradation of the sample decreased [34]. Compared with rice starch, the Mw/Mn of ERS and SPI-WPI-ERS increased, indicating that the aggregate state of starch was destroyed and the molecular distribution was broadened after extrusion treatment. Based on the above results, it was inferred that amylopectin with a large molecular size was more susceptible to degradation during extrusion.

### 3.3. XRD Analysis of RS, ERS, and SPI-WPI-ERS

**Figure 3:**
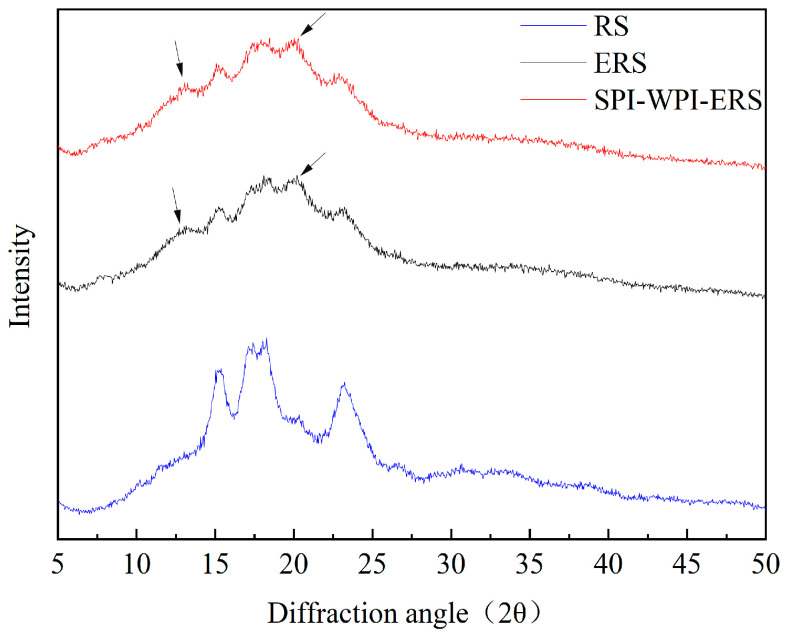
X-ray diffraction patterns of RS, ERS, and SPI-WPI-ERS.

The X-ray diffraction spectra of RS, ERS, and SPI-WPI-ERS are shown in Figure 3. The strong peaks in rice starch were observed at 15°, 17.2°, 17.9°, and 23°, which was an X-ray diagram of the A-type crystal structure. After extrusion, all the peaks gradually weakened in ERS and SPI-WPI-ERS, which indicated that most of the crystal structure of rice starch was destroyed. The clear new peaks were found at 13° and 20° in ERS and SPI-WPI-ERS, indicating the formation of V-type crystals after extrusion. It was speculated that protein–starch–lipid complexes were formed in SPI-WPI-ERS after extrusion.

Data on crystallinity of RS, ERS, and SPI-WPI-ERS are shown in Table 3. The crystallinity in the process of retrogradation was calculated using the XRD spectra. There was a negative relationship between crystallinity and retrogradation degree. The relative crystallinity of ERS and SPI-WPI-ERS was significantly lower than that of rice starch. Wu et al. [35] found that the relative crystallinity proportions of native corn starch (NS) and extruded corn starch (ES) were 12.32% and 8.95%, respectively, and the crystallinity of ES decreased significantly (*p* < 0.05). The crystalline region of the starch was damaged after extrusion, and ERS and SPI-WPI-ERS had more amorphous regions than rice starch. The crystallinity of SPI-WPI-ERS was lower than that of ERS, which was due to the cross-linking of starch molecules being inhibited by WPI and SPI during extrusion, the recrystallization of starch being hindered, and the formation rate of the ordered structure being reduced [36].

### 3.4. FTIR Spectra of RS, ERS, and SPI-WPI-ERS

**Figure 4:**
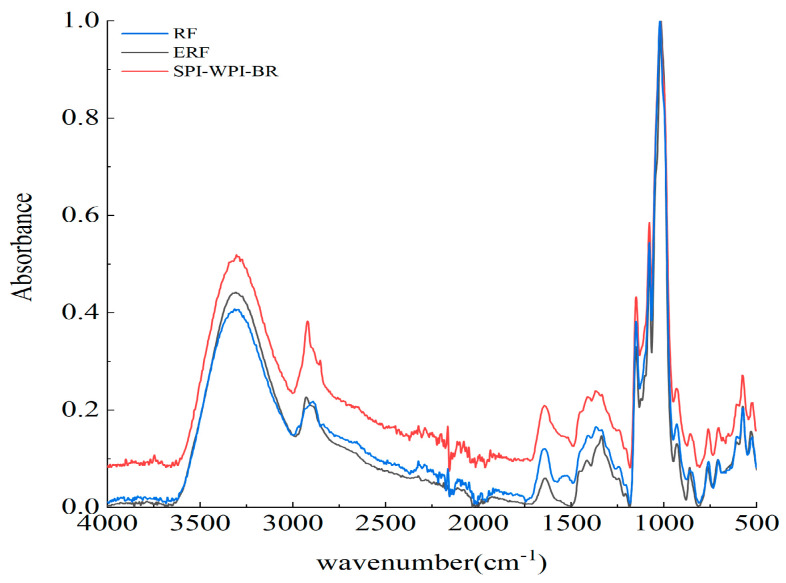
FTIR spectra (4000–500 cm^−1^) of RS, ERS, and SPI-WPI-ERS.

The infrared spectra of RS, ERS, and SPI-WPI-ERS are illustrated in Figure 4. The typical peaks of starch molecular absorption were 3400, 1700, 1370, 1014–1200, 861–764, and 576 cm^−1^. The short-range ordered structures of RS, ERS, and SPI-WPI-ERS were described by three characteristic peaks at 1047 cm^−1^, 1022 cm^−1^, and 995 cm^−1^. The absorbance at 1047 cm^−1^ was related to the ordered structure of starch, the absorbance at 1022 cm^−1^ was related to the amorphous structure of starch, and the absorbance at 995 cm^−1^ was associated with the hydrogen bond structure between starch molecules [37]. The positions and shapes of the absorption peaks of RS, ERS, and SPI-WPI-ERS were similar, and no new absorption peak appeared in the figure, indicating that the addition of WPI and SPI to rice flour and extrusion exerted no impact on the functional groups of rice starch.

The absorbance ratios of 1047/1022 cm^−1^ and 1022/995 cm^−1^ indicated the degree of order in the starch. The sample retrogradation became more difficult, accompanied by a decline in starch order [38]. The ratios of 1047/1022 cm^−1^ and 1022/995 cm^−1^ for ERS and SPI-WPI-ERS were lower than that for RS (Table 3). Zhang et al. [39] reported that the crystal structure of native rice starch (NRS) was destroyed by improved extrusion cooking technology (IECT), resulting in the transformation of ordered starch into amorphous starch and a decrease in the order state of NRS. Intriguingly, our experiment produced similar results. These results indicated that the ordered structure of starch was destroyed and the retrogradation of rice starch was inhibited by the gelatinization of starch molecules, which was caused by extrusion treatment. The ratios of 1047/1022 cm^−1^ and 1022/995 cm^−1^ for SPI-WPI-ERS were lower than those for ERS. This was due to the addition of WPI and SPI, further inhibiting the formation of the ordered structure [40].

### 3.5. SEM Analysis of RS, ERS, and SPI-WPI-ERS

**Figure 5:**
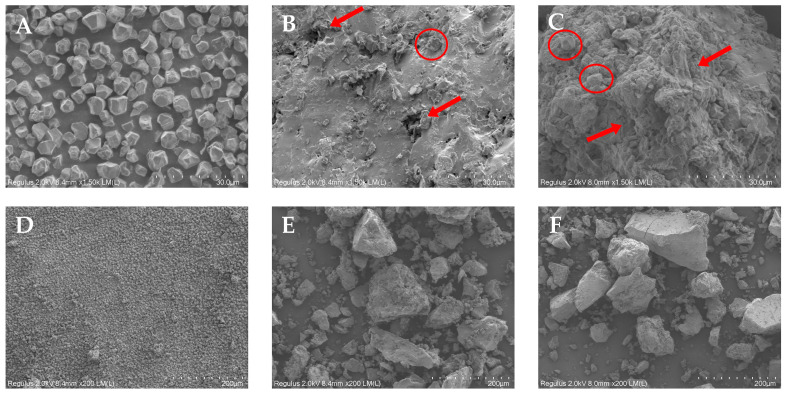
SEM of RS, ERS, and SPI-WPI-ERS. (**A**) RS (×1500); (**B**) ERS (×1500); (**C**) SPI-WPI-ERS (×1500); (**D**) RS (×200); (**E**) ERS (×200); and (**F**) SPI-WPI-ERS (×200).

The morphology of RS, ERS, and SPI-WPI-ERS is shown in Figure 5. A microscope was an important instrument for understanding the structure of the starch granules, and the changes in and replacement areas of the starch granules after extrusion were detected using SEM.

It can be seen from Figure 5A,D that the surface morphology of rice starch was polygonal, the edge was clear, all the natural starch granules were smooth, and there was no crack or fracture on the surface, which is the typical morphological structure of rice starch. It can be seen from Figure 5B,E that ERS became a continuous matrix formed by gelatinized starch, a small amount of pores and destroyed starch granules were observed, and intact starch granules almost completely disappeared. This was consistent with NRS treated using IECT by Zhang et al. [39] where a highly dense morphology and some cavities were observed on the surface of NRS. It can be seen from Figure 5C,F that, compared with ERS, the network structure of SPI-WPI-ERS was more compact, the pores were more uniform, the particle size was larger, and a small amount of starch granules could be observed. Chu et al. [41] also found that the microstructure of *Smilax china* L. starch–soy protein isolate (SCS-SPI) gel changed from a loose structure to a large piece with the addition of SPI. The network structure was rearranged and became denser and more regular. The addition of WPI to rice flour resulted in a very uniform and compact structure in the starch granules, which were deeply embedded in the protein network, as described by [42]. The results of Li et al. [43] showed that the structure of starch granules was destroyed by the thermal effect and shear force during extrusion, and a rougher and more irregular granule structure was observed. In summary, it could be speculated that the addition of WPI and SPI reduces the decomposition of starch by extrusion, inhibits the cross-linking and recrystallization between starch molecules, and improves the anti-retrogradation capability of the starch molecules.

### 3.6. PLM Analysis of RS, ERS, and SPI-WPI-ERS

There were a crystalline structure and amorphous structure in the interior of the starch granules, which were different in density and refractive index, resulting in anisotropy. Thus, the starch granules could be divided into four white areas under the polarizing microscope, and a black polarization cross could be observed at its umbilical point. When the molecular arrangement in the crystalline region of the starch granules was disturbed, the brightness of the crystalline region transmitted by polarized light would become dark, and then the polarized light cross would weaken or even disappear [44]. Therefore, a change in the polarization cross could directly reflect the alteration in the crystalline structure of the starch granules.

**Figure 6:**
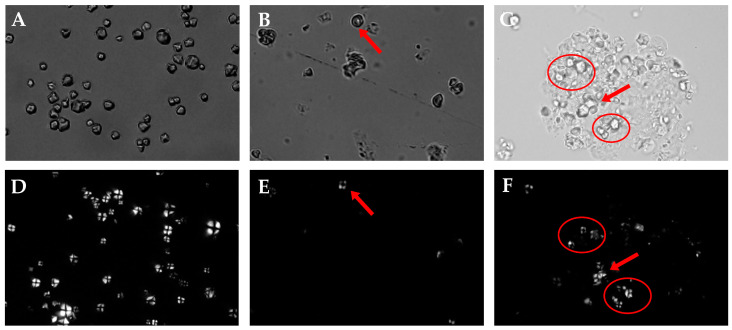
PLM of RS, ERS, and SPI-WPI-ERS. (**A**) RS taken under natural light; (**B**) ERS taken under natural light; (**C**) SPI-WPI-ERS taken under natural light; (**D**) RS taken under polarized light; (**E**) ERS taken under polarized light; and (**F**) SPI-WPI-ERS taken under polarized light.

The polarization crosses of RS, ERS, and SPI-WPI-ERS are shown in Figure 6. It can be seen from Figure 6A,D that the rice starch had a clear and bright polarization cross, indicating that the starch granules had a complete crystal structure [45]. Figure 6B,E shows that only a few indistinct polarization crosses were observed in ERS, and the fission of most starch granules resulted in dim polarization crosses. Figure 6C,F shows that most broken starch granules were observed in SPI-WPI-ERS, the edges of some granules became blurred and swollen, and the polarization cross became dark or disappeared, which was not a complete cross-shaped structure. This phenomenon was due to the fact that the crystal structure of the rice starch granules was damaged by extrusion, which was beneficial for water molecules to enter the starch granules, gelatinize, and expand [46]. The addition of WPI and SPI weakened the damage to rice starch by extrusion. The results of Li et al. [47] showed that the brightness of Maltese crosses in extrudates was lower than that in native starch granules, and some native starch granules were broken into smaller granules.

### 3.7. WAI and WSI Analysis of RS, ERS, and SPI-WPI-ERS

**Figure 7:**
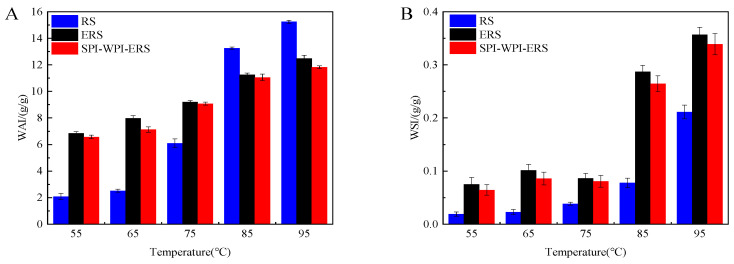
(**A**) WAI of RS, ERS, and SPI-WPI-ERS; and (**B**) WSI of RS, ERS, and SPI-WPI-ERS.

The WAI and WSI of RS, ERS, and SPI-WPI-ERS at different temperatures are shown in Figure 7. WAI and WSI reflect the changes in starch granules and starch chains during starch gelatinization.

After extrusion treatment, the intermolecular and intramolecular hydrogen bonds of rice starch were destroyed. Then, the crystal structure of rice starch was damaged to varying degrees, the structure became loose, and more hydroxyl groups were exposed to form hydrogen bonds with water, so Figure 7A shows that the WAIs of ERS and SPI-WPI-ERS were much higher than that of rice starch at 55 °C, 65 °C, and 75 °C. However, at 85 °C and 95 °C, the starch was completely gelatinized, and the WAIs of ERS and SPI-WPI-ERS were less than that of rice starch because of the degradation of modified starch molecules and the weakening of water holding capacity. Mesa et al. [48] found that the WAI of native corn starch–soy protein concentrate (CS-SPC) extrudates decreased with an increased content of soy protein concentrate. It can be seen from Figure 7B that the WSIs of ERS and SPI-WPI-ERS were much higher than that of rice starch, and the increase in WSI was mainly due to the degradation of starch molecules. When ERS and SPI-WPI-ERS were destroyed, amylopectin and amylose were more easily extruded. After adding different concentrations (16%, 32%, and 40%) of WPI to corn starch, the starch molecules were destroyed or gelatinized, leading to an increase in WSI [49] The WAI and WSI of SPI-WPI-ERS were lower than that of ERS, which might have been due to the surface of the starch granules being covered or enwrapped by the WPI and SPI formed films during extrusion, which prevented the starch from contacting with water, regulated the swelling and dissolution of starch granules, and delayed the gelatinization of starch [50].

### 3.8. RVA Analysis of RS, ERS, and SPI-WPI-ERS

**Figure 8:**
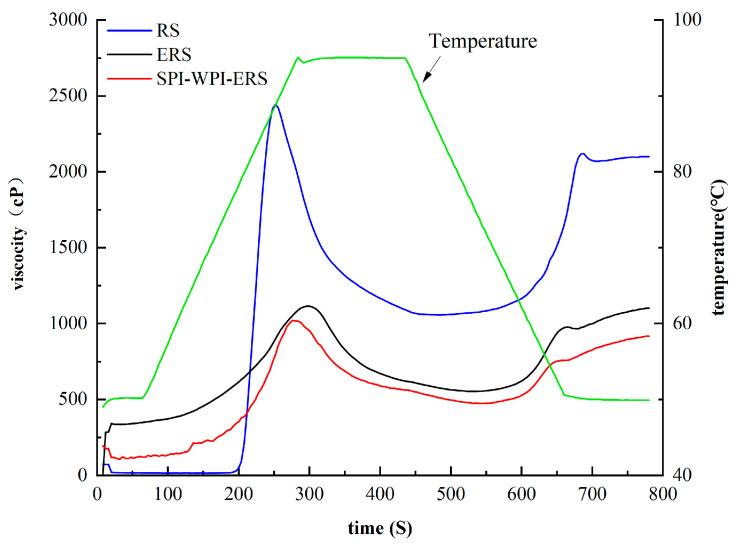
Viscosity change curves of RS, ERS, and SPI-WPI-ERS.

The viscosity curves of RS, ERS, and SPI-WPI-ERS are shown in Figure 8. The peak viscosity (PV) is the viscosity of the starch granules when they absorbed water and could swell. Setback (SB) is the difference between the peak viscosity (PV) and the trough viscosity (TV), which indicates the setback degree of starch during the cooling process. Break down (BD) viscosity is the difference between the PV and final viscosity (FV), indicating the stability and shear resistance of starch paste. The smaller BD values indicated the better thermal stability and the stronger shear resistance of the samples [19].

The pasting properties of starches in RS, ERS, and SPI-WPI-ERS are listed in Table 4. The PV, TV, BD, FV, and SB of ERS and SPI-WPI-ERS were lower than those of rice starch. A starch–lipid complex was formed in the process of extrusion, the crystalline structure of the rice starch granules was destroyed, and the recombination of starch was affected by the formation of small molecular polysaccharides. Thus, starch retrogradation and disintegration were inhibited. The above results are consistent with the data of the XRD and FTIR analyses. The PV, TV, BD, FV, and SB of SPI-WPI-ERS were lower than those of ERS, as the protein was wrapped around the starch granules in the extrusion process, and the protein and the starch granules were attracted to each other through electrostatic interaction, so the formation of an ordered structure of the starch was limited and the recrystallization of starch was inhibited [35]. It was inferred that the starch was not completely gelatinized during extrusion, as water in the sample was competed by starch and protein. The limitation of water resulted in a significant difference in gelatinization parameters after extrusion. This conclusion was consistent with the results of Fernández-Gutiérreza et al. [51], who found that a weak and less stable gel in a starch–casein complex was formed after extrusion, resulting in a decrease in both the setback and the viscosity value. Valle et al. [52] revealed that the low viscosity of amylopectin was attributed to the presence of short-chain branches in its macromolecular structures, which is consistent with the results in Table 1 and Table 4.

### 3.9. Rheological Properties of RS, ERS, and SPI-WPI-ERS

**Figure 9:**
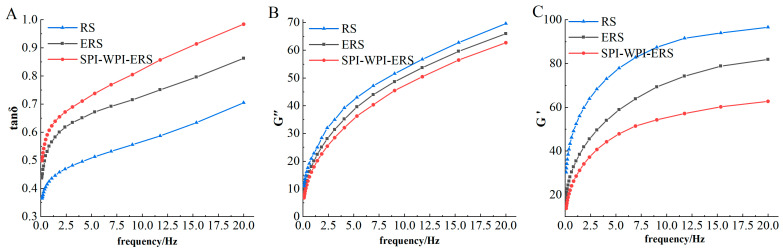
Dynamic rheological trends of RS, ERS, and SPI-WPI-ERS. (**A**) Tanδ of RS, ERS, and SPI-WPI-ERS; (**B**) G″ of RS, ERS, and SPI-WPI-ERS; and (**C**) G′ of RS, ERS, and SPI-WPI-ERS.

The rheological results of RS, ERS, and SPI-WPI-ERS are displayed in Figure 9. The storage modulus (G′) reflects the ability of the starch paste to recover its original shape after deformation, corresponding to the rigidity and elasticity of the gel. The loss modulus (G″) reflects the resistance of the starch paste to flow and corresponds to the viscosity and flow of the gel. Tanδ is the ratio of the loss modulus to the storage modulus (G″/G′). 

It can be seen from Figure 9B,C that the G′ and G″ of RS, ERS, and SPI-WPI-ERS increased with an increase in frequency, and G′ was higher than G″. The G′ and G″ of ERS and SPI-WPI-ERS were lower than those of rice starch. It can be seen from Figure 9A that the tanδ values of RS, ERS, and SPI-WPI-ERS were less than 1, and the tanδ value of ERS and SPI-WPI-ERS were higher than that of rice starch, indicating the transition of ERS and SPI-WPI-ERS towards the liquid characteristic. This fact was due to the degradation of the starch molecules into fragments of a smaller molecular weight, inhibiting the rearrangement of starch molecules in the retrogradation process and resulting in a decrease in the G′ and G″ of modified starch. The G′ and G″ of RS, ERS, and SPI-WPI-ERS were decreased with the addition of WPI and SPI. It might be that the starch granules were coated with proteins as a continuous matrix, resulting in more starch becoming gelatinized and aggregating with WPI and SPI. The relatively stable network structure of protein could not be formed, so the rheological property of the starch was influenced, the viscosity and elasticity of the formed protein–starch polymer were reduced, and the hydrogen bond force was relatively weak; gels tended to be more fluid [53]. This was similar to the results of Chu et al. [41], who found that the G′ and G″ of SCS-SPI gel decreased, but tanδ increased with a decrease in SCS concentration.

### 3.10. Thermal Properties of RS, ERS, and SPI-WPI-ERS

The results of the thermodynamic properties of RS, ERS, and SPI-WPI-ERS are shown in Table 5. Differential scanning calorimetry is a common thermodynamic analysis method which can be used to predict the cooking properties of rice products. Gelatinization temperature and enthalpy (∆H) reflect the degree of gelatinization of starch granules [54]. A higher gelatinization temperature and ∆H indicated that a higher temperature for the destruction of the crystalline region of starch molecules needed to be maintained, and starch was more prone to retrogradation [55].

It can be seen from the table that the onset (T_0_), peak (T_P_), and conclusion (Tc) gelatinization temperatures, ∆H, and phase transition temperature (∆T) of ERS and SPI-WPI-ERS were significantly lower than those of rice starch. The double helix structure of the starch molecules was destroyed and the crystalline lamellae were melted in the process of extrusion, so only a small amount of energy was needed for the phase transition of RS, ERS, and SPI-WPI-ERS [56,57]. However, there was no significant difference between ERS and SPI-WPI-ERS, indicating that the thermodynamic properties of starch were not significant with the addition of exogenous protein. Luo et al. [58] studied the synergistic effect of vegetable protein hydrolysate and xanthan gum on the short-term and long-term retrogradation of rice starch. The results showed that the ∆H of natural starch decreased with the addition of hydrocolloid and rice protein.

## 4. Conclusions

The retrogradation behavior of rice starch when adding exogenous protein and using extrusion technology was investigated by molecular weight, amylopectin branch length distribution, total and amylose content, WAI, WSI, thermal properties, microstructure, rheological properties, XRD, RVA, and FTIR. The results of the RVA, thermal properties, and rheological properties revealed that the swelling of the starch granules during extrusion was inhibited by the addition of SPI and WPI, and the ∆H and retrogradation rate of rice starch were decreased. During the process of extrusion, the microstructure of rice starch was destroyed, the molecular weight was decreased, and the long amylopectin was degraded into amylose. The rearrangement of starch molecules in the retrogradation process was inhibited and the setback was reduced. The XRD and FTIR results also supported these observations. The crystallinity of ERS and SPI-WPI-ERS decreased by 26.07% and 55.72%, respectively. The setback of ERS and SPI-WPI-ERS decreased by 47.46% and 57.53%, respectively. The enthalpy values of ERS and SPI-WPI-ERS decreased by 24.87% and 33.73%, respectively. These results demonstrated that blends of SPI, WPI, and rice flour were extruded, which resulted in the effective inhibition of the retrogradation of rice starch. Hence, a good method for preparing rice food with a low rate of retrogradation was proposed, and more rice food satisfying the living needs of people was prepared.

## Figures and Tables

**Table 1:** Amylopectin branch chain length distribution of RS, ERS, and SPI-WPI-ERS.

Samples	A: 6 ≤ DP ≤ 12	B_1_: 13 ≤ DP ≤ 24	B_2_: 25 ≤ DP ≤ 36	B_3_: 37 ≤ DP
RS	26.63 ± 0.24 ^b^	49.97 ± 0.07 ^c^	11.18 ± 0.16 ^a^	11.54 ± 0.27 ^a^
ERS	27.32 ± 0.16 ^a^	50.82 ± 0.35 ^a^	11.09 ± 0.06 ^c^	11.45 ± 0.22 ^b^
SPI-WPI-ERS	26.73 ± 0.39 ^b^	50.56 ± 0.28 ^b^	11.15 ± 0.11 ^b^	11.53 ± 0.32 ^a^

Different lower-case letters in the same column were significantly different (*p* < 0.05).

**Table 2:** Total starch, amylose content, and molecular weight of RS, ERS, and SPI-WPI-ERS.

Samples	Total Starch (%)	Amylose Content (%)	Mn (kDa)	Mw (kDa)	Mw/Mn
RS	77.40 ± 0.16 ^a^	17.10 ± 0.10 ^c^	26691.42 ± 26.16 ^a^	72246.39 ± 9.01 ^a^	2.71 ± 0.12 ^c^
ERS	74.00 ± 0.61 ^c^	21.29 ± 0.19 ^a^	20139.40 ± 14.64 ^b^	64082.54 ± 25.32 ^c^	3.18 ± 0.06 ^b^
SPI-WPI-ERS	75.78 ± 0.53 ^b^	18.12 ± 0.17 ^b^	20286.12 ± 10.58 ^b^	67605.68 ± 27.53 ^b^	3.33 ± 0.19 ^a^

Different lower-case letters in the same column were significantly different (*p* < 0.05).

**Table 3:** Infrared (IR) peak ratio, crystal pattern, and crystallinity of RS, ERS, and SPI-WPI-ERS.

Samples	Crystal Pattern	Degree of Crystallinity (%)	IR Peak Ratio of 1047/1022 cm^−1^	IR Peak Ratio of 1022/995 cm^−1^
RS	A	40.58 ± 0.24 ^a^	0.7375 ± 0.17 ^a^	1.24 ± 0.02 ^a^
ERS	V	30.00 ± 0.15 ^b^	0.7229 ± 0.15 ^b^	1.21 ± 0.01 ^b^
SPI-WPI-ERS	V	17.97 ± 0.11 ^c^	0.6873 ± 0.08 ^c^	1.20 ± 0.00 ^c^

Different lower-case letters in the same column were significantly different (*p* < 0.05).

**Table 4:** Pasting properties of RS, ERS, and SPI-WPI-ERS.

Samples	PV (cP)	TV (cP)	BD (cP)	FV (cP)	SB (cP)
RS	2444 ± 31.36 ^a^	1056 ± 10.56 ^a^	1388 ± 12.43 ^a^	2099 ± 32.12 ^a^	1043 ± 13.09 ^a^
ERS	1115 ± 6.35 ^b^	553 ± 5.87 ^b^	562 ± 6.24 ^b^	1101 ± 10.33 ^b^	548 ± 6.27 ^b^
SPI-WPI-ERS	1021 ± 7.84 ^c^	474 ± 5.30 ^c^	547 ± 8.16 ^c^	917 ± 9.17 ^c^	443 ± 3.15 ^c^

Different lower-case letters in the same column were significantly different (*p* < 0.05).

**Table 5:** Thermal properties of RS, ERS, and SPI-WPI-ERS.

Samples	T_0_ (°C)	T_p_ (°C)	T_c_ (°C)	∆H (J/g)	∆T (°C)
RS	38.82 ± 1.01 ^a^	96.66 ± 0.88 ^a^	157.23 ± 5.16 ^a^	276.25 ± 5.16 ^a^	118.41 ± 2.55 ^a^
ERS	37.15 ± 1.00 ^b^	91.67 ± 1.07 ^b^	143.24 ± 5.65 ^b^	207.55 ± 2.50 ^b^	106.09 ± 4.51 ^b^
SPI-WPI-ERS	35.19 ± 0.99 ^c^	90.05 ± 1.20 ^c^	140.48 ± 7.14 ^c^	183.08 ± 5.12 ^c^	105.29 ± 4.50 ^b^

Different lower-case letters in the same column were significantly different (*p* < 0.05).

## Data Availability

The original contributions presented in the study are included in the article, further inquiries can be directed to the corresponding author.
